# Identification of the Predictive Models for the Treatment Response of Refractory/Relapsed B-Cell ALL Patients Receiving CAR-T Therapy

**DOI:** 10.3389/fimmu.2022.858590

**Published:** 2022-03-17

**Authors:** Jingxian Gu, Sining Liu, Wei Cui, Haiping Dai, Qingya Cui, Jia Yin, Zheng Li, Liqing Kang, Huiying Qiu, Yue Han, Miao Miao, Suning Chen, Shengli Xue, Ying Wang, Zhengming Jin, Xiaming Zhu, Lei Yu, Depei Wu, Xiaowen Tang

**Affiliations:** ^1^ National Clinical Research Center for Hematologic Diseases, Jiangsu Institute of Hematology, The First Affiliated Hospital of Soochow University, Suzhou, China; ^2^ Institute of Blood and Marrow Transplantation, Collaborative Innovation Center of Hematology, Soochow University, Suzhou, China; ^3^ Key Laboratory of Thrombosis and Hemostasis of Ministry of Health, Institute of Blood and Marrow Transplantation, Suzhou, China; ^4^ Research and Development Department, Shanghai Unicar-Therapy Bio-Medicine Technology Co., Ltd., Shanghai, China

**Keywords:** chimeric antigen receptor T cells, refractory or relapsed B-cell acute lymphoblastic leukemia, predictive models, complete remission, MRD-negative complete remission

## Abstract

**Background/Aims:**

Chimeric antigen receptor (CAR) T cells for refractory or relapsed (r/r) B-cell acute lymphoblastic leukemia (ALL) patients have shown promising clinical effectiveness. However, the factors impacting the clinical response of CAR-T therapy have not been fully elucidated. We here aimed to identify the independent factors of CAR-T treatment response and construct the models for predicting the complete remission (CR) and minimal residual disease (MRD)-negative CR in r/r B-ALL patients after CAR-T cell infusion.

**Methods:**

Univariate and multivariate logistic regression analyses were conducted to identify the independent factors of CR and MRD-negative CR. The predictive models for the probability of remission were constructed based on the identified independent factors. Discrimination and calibration of the established models were assessed by receiver operating characteristic (ROC) curves and calibration plots, respectively. The predictive models were further integrated and validated in the internal series. Moreover, the prognostic value of the integration risk model was also confirmed.

**Results:**

The predictive model for CR was formulated by the number of white blood cells (WBC), central neural system (CNS) leukemia, *TP53* mutation, bone marrow blasts, and CAR-T cell generation while the model for MRD-negative CR was formulated by disease status, bone marrow blasts, and infusion strategy. The ROC curves and calibration plots of the two models displayed great discrimination and calibration ability. Patients and infusions were divided into different risk groups according to the integration model. High-risk groups showed significant lower CR and MRD-negative CR rates in both the training and validation sets (*p* < 0.01). Furthermore, low-risk patients exhibited improved overall survival (OS) (log-rank *p* < 0.01), higher 6-month event-free survival (EFS) rate (*p* < 0.01), and lower relapse rate after the allogeneic hematopoietic stem cell transplantation (allo-HSCT) following CAR-T cell infusion (*p* = 0.06).

**Conclusions:**

We have established predictive models for treatment response estimation of CAR-T therapy. Our models also provided new clinical insights for the accurate diagnosis and targeted treatment of r/r B-ALL.

## Introduction

Refractory or relapsed (r/r) B-cell acute lymphoblastic leukemia (ALL) remains one of the most fatal hematological malignancies with the reported median overall survival (OS) ranging from 3.0 to 8.4 months even after salvage chemotherapy or transplantation (1–4). Chimeric antigen receptor (CAR) T-cell therapy emerges as a new cellular immunotherapeutic strategy these years showing impressive efficacy in r/r B-ALL (5, 6). Published clinical trials have indicated a high complete remission (CR) rate of 67% to 93% in r/r B-ALL patients who received CAR-T cell infusion (7–13). However, there are still approximately 10% to 30% patients who had no response to the treatment and a large proportion of patients who relapsed soon after achieving CR (7–13). This is mainly because engineered T-cell therapy is an immensely individualized treatment due to the high heterogeneity of the malignancies, patients, and these functional T cells, which was originally collected from each patient (6, 14). Hence, to further improve therapeutic response, it is imperative for effective predictive tools to select the most benefited patients before infusion. In addition, on account of the considerable costs of CAR-T therapy, pretreatment evaluation is also of great significance to improve the cost-effectiveness as the application of this therapy is increasingly broadened (15, 16).

The influence factors of CAR-T therapeutic effect remain unclear. The results of several studies trying to identify these clinical factors varied (17–19). Besides, previous studies mainly centered on the resistance of cancer cells to the modified T cells and the lack of the persistence of CAR-T cells, irrespective of the clinical characteristics of the patients themselves (20, 21). Therefore, studies investigating the baseline characteristics as the potential predictive or prognostic factors of this novel therapy are warranted to help facilitate clinical prediction and guide personalized treatment. Here, we enrolled a large cohort of r/r B-ALL patients treated with CAR-T cells from three clinical trials and performed analyses to screen the independent factors of CAR-T treatment response. Also ultimately, in this study we first established the simple-to-use models for predicting the clinical outcome of these patients.

## Materials and Methods

### Patients and Data Collection

A total of 286 consecutive r/r B-ALL patients who received CAR-T cells enrolled on three clinical trials (www.clinicaltrials.gov, identifiers: NCT03919240, NCT03614858, and NCT03275493) from December 2015 to September 2021 were included in this study. The major inclusion criteria were as follows: (1) diagnosed as refractory/relapsed B-ALL; (2) Karnofsky performance status score ≥ 60 or Eastern Cooperative Oncology Group performance score ≤ 2; (3) estimated survival time ≥3 months; and (4) ineligible for or refusal to allogeneic hemopoietic stem cell transplantation (allo-HSCT). Relapsed disease was defined as >5% bone marrow blasts, reappearance of circulating blasts, or development of extramedullary disease. Refractory disease was defined as those patients who did not achieve CR after 2 courses of intensive induction chemotherapy (13, 22). All three clinical trials and this study were approved by the Institutional Ethics Committees of the First Affiliated Hospital of Soochow University and conformed to the provisions of the Declaration of Helsinki. Written informed consent was obtained from the patients or their legal guardians. Clinical data including the clinical characteristics of every subject and the baseline information of the CAR-T cells used for each time of infusion for each patient were collected. All the data were extracted from the electronic medical record system of the patients.

### Study Design

**Figure 1:**
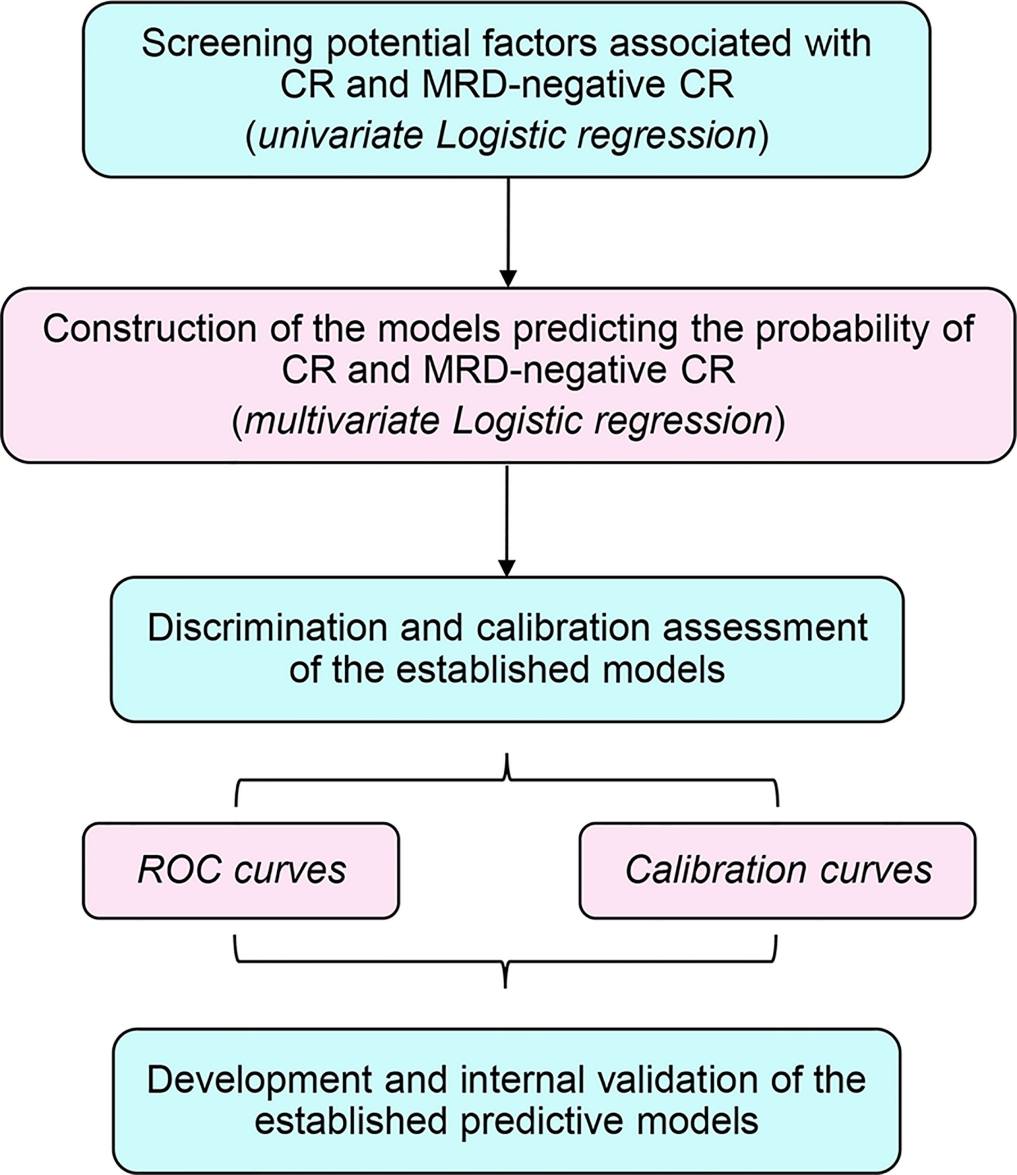
Flowchart of the analytic process of this study.

The overall design of this study is as summarized in **Figure 1**. Herein, two main phases including constructing and validating the predictive models for r/r B-ALL patients after CAR-T therapy were conducted. Out of all the 286 subjects, 204 patients enrolled on the above three clinical trials from December 2015 to March 2020 were treated as the discovery dataset. Another 82 patients from April 2020 to September 2021 were taken as the independent validation set. During the discovery phase, the first time of CAR-T cell infusion of each subject from the discovery set was included for the model construction. Firstly, the univariate and multivariate logistic regression analyses were applied to screen and identify the independent CR- and minimal residual disease (MRD)-negative CR-related factors. Except binary variables, all the variables were transformed into categorical ones before putting into logistic models. Moreover, the cutoff values adopted for the transformed covariates were as shown in **Table 1**. In the meantime, the predictive models were generated from the forward stepwise multivariate analysis (likelihood ratio). Then, for model evaluation, receiver operating characteristic (ROC) curves, along with the corresponding area under the curve (AUC), were used to assess the discrimination ability. The calibration curves for estimation of the consistency between the actual observation and the model-predicted value, and the Hosmer–Lemeshow chi-square (χ^2^) test were carried out simultaneously for evaluating the accuracy and goodness of fit (23). In the validation analyses, the two models predicting CR and MRD-negative CR were integrated into one risk model for better prediction. First of all, we used all the infusions of 204 patients in the discovery set (some patients received more than one time of CAR-T cell infusion) as the training set to preliminarily validate the risk model. Furthermore, we also followed up the 204 participants for their survival status, 6-month event-free survival (EFS) status, subsequent treatments including transplantation, etc. The OS, 6-month EFS rate, and relapse rate of those who received allo-HSCT after CAR-T therapy were compared between different risk groups, respectively, to confirm the prognostic value of the modified model. Eventually, all the infusions of an independent patient cohort were used to further validate our risk model.

**Table 1:** Patient covariates (N = 204).

Covariates	n*
*Baseline characteristics*	
Gender (female/male)	105/99
Age (years)	30 (6–65)
WBC^a^ (×10^9^/L) (<20/20–100/≥100)	110/52/42
Extramedullary disease^#^ (yes/no)	62/142
CNS leukemia (yes/no)	14/190
High cytogenetic risk	
Ph+ (yes/no)	63/141
Ph-like (yes/no)	14/190
TP53 mutation (yes/no)	16/188
T315I mutation (yes/no)	28/176
Del (7) (yes/no)	8/196
Complex karyotype (yes/no)	48/156
MLL aberrations (yes/no)	12/192
WT1 (positive/negative ^b^)	65/139
EVI1 (positive/negative ^b^)	36/168
IgH rearrangement (yes/no)	8/196
TCR rearrangement (yes/no)	5/199
Switched from CML lymphoblastic crisis (yes/no)	10/194
Disease status (relapsed/refractory)	159/45
Response after first chemotherapy (CR/PR/NR)	159/16/29
Previous allo-HSCT (yes/no)	44/160
Number of relapses (0/1/>1)	42/113/49
Number of previous therapies (<3/3–4/>4)	56/76/72
*CAR-T therapy*	
Bone marrow blasts (%)^c^ (<5/5–25/25–50/>50)	99/39/31/35
Lymphodepletion regimen (FC/without FC)	193/11
Decitabine (yes/no)	12/192
Rituximab (yes/no)	12/192
MVP (yes/no)	19/185
Infusion strategy (single target/dual-target/sequential infusion)	152/42/10
IL-6 knockdown (yes/no)	11/193
Generation (2nd/3rd or 4th)	151/53
Interferon (yes/no)	9/195
Glucocorticoid (yes/no)	45/159
Tocilizumab (yes/no)	26/178
CRS after CAR-T	
CRS grade (0–2/>2)	153/51
CRES (0–2/>2)	187/17
Hemophagocytic histiocytosis (yes/no)	4/200
Tumor lysis syndrome (yes/no)	3/201

WBC, white blood cells; CNS, central nervous system; Ph, Philadelphia chromosome; CML, chronic myeloid leukemia; CR, complete remission; PR, partial remission; NR, no response; allo-HSCT, allogeneic hematopoietic stem cell transplantation; CAR-T, chimeric antigen receptor T-cell immunotherapy; FC, fludarabine and cyclophosphamide; MVP, mitoxantrone, vincristine and prednisone; CRS, cytokine release syndrome; CRES, CAR-T cell-related encephalopathy syndrome.

*Median and range for age, absolute patient numbers for other covariates.

^#^EMD other than CNS involvement.

^a^The numbers of WBC in peripheral blood were detected when newly diagnosed.

^b^Positive: copy number>10/10,000abl copies, otherwise negative.

^c^Bone marrow blasts detected before lymphodepletion or CAR-T cell infusion (for those without lymphodepletion).

### CAR-T Therapy

CAR-T cells were produced by Shanghai UniCAR Technology Co., Ltd. (UCT, Shanghai, China) (24, 25). Briefly, mononuclear cells for CAR-T cell production were obtained from the peripheral blood of the patients, transplant, or healthy donors by leukapheresis. Then, these cells were purified and transduced with lentiviral vector encoding chimeric T-cell antigen receptors. The CAR was finally composed of targeted single-chain variable fragment (scFv), intracellular domain including 4-1BB or CD28 as a co-stimulation signal, and a cytoplasmic signaling sequence, CD3ζ. Antigen receptors of these CAR-T cells contain CD19, CD22, and dual target, CD19+CD22.

Patients received fludarabine and cyclophosphamide (FC)-based lymphodepletion regimen prior to CAR-T cell infusion. Bridging chemotherapy before lymphodepletion included decitabine, rituximab, or other cytotoxic chemotherapy, such as MVP regimen (MVP: mitoxantrone, vincristine and prednisone). For one single time of infusion, CAR-T cells were infused on 3 consecutive days with 10%, 30%, and 60% of the total dose, respectively, or on 2 days, 40% for day +1 and 60% for day +2. The median cell dose of the infused cells was 0.5 × 10^7^/kg (range, 0.05–67 × 10^7^/kg). Philadelphia chromosome (Ph)-positive B-ALL patients were given tyrosine kinase inhibitors (TKI).

### Response Assessment and Follow-Up

CR was defined as <5% blasts in bone marrow and absence of extramedullary disease. MRD-negative CR was defined as <0.01% blasts in bone marrow detected by multicolor flow cytometry and, also, no evidence of extramedullary disease (13, 19). Bone marrow examination was performed at least 28 days after CAR-T cell infusion for evaluation of treatment response. Cytokine release syndrome (CRS) including CAR-T cell-related encephalopathy syndrome (CRES) was graded according to the criteria proposed by Lee et al. (26, 27). The last follow-up of the long-term survival of the patients from the discovery group was on December 31, 2020. OS was defined as the interval between the date of the first infusion and the date of death of any cause, allotransplant, or the last follow-up. EFS was calculated from the date of the first infusion to the date of relapse, death, or the last follow-up. If a patient had no response to CAR-T therapy, EFS status was also defined as 1 (28).

### Statistical Considerations

To prevent missing more clinically significant indexes, the *p* values less than 0.2 from univariate analysis were considered as the threshold for inclusion in multivariate analysis. The univariate and multivariate logistic regression analyses were carried out *via* SPSS 23.0 for Windows (SPSS, Chicago, IL). The ROC curves with AUC calculation, Kaplan–Meier curves, and log-rank tests were performed in GraphPad Prism, version 7.0. The Hosmer–Lemeshow test was conducted by SPSS software (version 23.0), and its *p* value more than 0.05 indicated that the difference between the expected and actually observed values was insignificant. For each infusion, the patient could obtain a probability score of CR or MRD-negative CR generated from the constructed models. The clinical outcome of each infusion was then evaluated as high probability of CR (X^high^) or low probability of CR (X^low^), and high probability of MRD-negative CR (Y^high^) or low probability of MRD-negative CR (Y^low^) based on the cutoff values generated from ROC curves when Youden’s indexes achieved maximum. The proportions of CR, MRD-CR, event-free, and relapse patients among different groups were compared by χ^2^ test or Fisher’s exact test using absolute numbers of subjects in SPSS software as well. *p* < 0.05 in this study was considered statistically significant, otherwise indicated.

## Results

### Clinical Characteristics of the Study Population

The baseline characteristics of 204 participants in the discovery set and the CAR-T cells they initially received are presented in **Table 1**. Out of them, 10 (4.9%) r/r B-ALL patients were switched from CML and 44 (21.6%) had at least one allo-HSCT before CAR-T therapy. The Philadelphia chromosome was detected positive in 63 (30.9%) samples. 42 (20.6%) participants had extremely high tumor burden with white blood cells (WBC) in peripheral blood more than 100 × 10^9^/l when newly diagnosed, while nearly half of the patients (N = 99) had relatively low (<5% of blasts) tumor burden before lymphodepletion, because most of the patients were heavily treated before CAR-T infusion with a median previous therapy number of 4, leading to varying degrees of bone marrow hypocellularity. Extramedullary disease other than CNS involvement was found in 62 (30.4%) subjects at enrollment. 14 patients were diagnosed with central neural system (CNS) leukemia. CRS occurred in 155 (76.0%) patients including 51 (25.0%) with severe symptoms (grade ≥3). 17 (8.3%) patients developed CRES of grade 3 or higher. In all, 176 (86.3%) patients achieved CR after CAR-T therapy and among them, 145 (71.1%) achieved MRD-negative CR. The 1-year probability of survival was 71.8% with the median follow-up time of 16.2 months (range: 1.3–52.1 months).

### Searching for the Factors Associated With CR and MRD-Negative CR in r/r B-ALL Patients After CAR-T Therapy

To search for the possible factors of r/r B-ALL patients receiving CAR-T treatment, we first conducted univariate logistic regression analysis on clinical characteristics of the patients (**Table 2**) and the CAR-T cells infused for the first time (**Table 3**). The univariate analytic results revealed that the number of WBC in peripheral blood detected at diagnosis, CNS leukemia, *TP53* mutation, number of relapses, and bone marrow blasts detected before lymphodepletion or CAR-T cell infusion (for those without lymphodepletion) were significantly associated with CR after CAR-T cell infusion (*p* < 0.05, **Tables 2**, **3**). Also, two factors, number of relapses and CRS grade, were shown to have significant relations to MRD-negative CR (*p* < 0.05, **Tables 2**, **3**), while the relation between CRS grade and CR was marginally significant (*p* = 0.06, **Table 3**). The following factors, the number of WBC in peripheral blood detected at diagnosis (*p* = 0.09, **Table 2**) and bone marrow blasts detected before lymphodepletion or CAR-T cell infusion (*p* = 0.08, **Table 3**), were found to have marginally significant correlations with MRD-negative CR despite their statistically significant relations to CR.

**Table 2:** Univariate logistic regression analyses of the clinical characteristics of r/r B-ALL patients associated with CR and MRD-negative CR.

Variables	CR		MRD-negative CR	
	OR (95% CI)	*p* value	OR (95% CI)	*p* value
Gender				
Female	1.00 (reference)		1.00 (reference)	
Male	0.56 (0.25–1.27)	0.17*	1.17 (0.64–2.14)	0.61
Age (years)		0.39		0.62
<20	1.00 (reference)		1.00 (reference)	
20–40	0.36 (0.10–1.33)	0.13*	0.69 (0.31–1.56)	0.38
40–60	0.35 (0.09–1.34)	0.12*	0.67 (0.28–1.60)	0.37
≥60	0.20 (0.02–2.55)	0.22	0.30 (0.04–2.36)	0.25
WBC^a^ (×10^9/L)		0.01*		0.09*
<20	1.00 (reference)		1.00 (reference)	
20–100	0.77 (0.26–2.24)	0.63	1.02 (0.48–2.19)	0.95
≥100	0.25 (0.10–0.64)	<0.01*	0.46 (0.22–0.96)	0.04*
Extramedullary disease^#^				
No	1.00 (reference)		1.00 (reference)	
Yes	1.71 (0.66–4.46)	0.27	1.41 (0.71–2.78)	0.32
CNS leukemia				
No	1.00 (reference)		1.00 (reference)	
Yes	0.25 (0.08–0.80)	0.02*	0.72 (0.23–2.23)	0.56
Ph+				
No	1.00 (reference)		1.00 (reference)	
Yes	1.14 (0.47–2.74)	0.78	1.15 (0.59–2.23)	0.68
Ph-like				
No	1.00 (reference)		1.00 (reference)	
Yes	2.15 (0.27–17.14)	0.47	1.76 (0.57–5.45)	0.32
*TP53* mutation				
No	1.00 (reference)		1.00 (reference)	
Yes	0.22 (0.07–0.67)	0.01*	0.49 (0.17–1.39)	0.18*
T315I mutation				
No	1.00 (reference)		1.00 (reference)	
Yes	0.69 (0.24–2.00)	0.50	0.69 (0.30–1.61)	0.40
Del (7)				
No	1.00 (reference)		1.00 (reference)	
Yes	1.12 (0.13–9.45)	0.92	1.23 (0.24–6.28)	0.80
Complex karyotype				
No	1.00 (reference)		1.00 (reference)	
Yes	0.60 (0.25–1.43)	0.25	0.98 (0.48–2.01)	0.97
*MLL* aberrations				
No	1.00 (reference)		1.00 (reference)	
Yes	1.80 (0.22–14.51)	0.58	1.24 (0.32–4.73)	0.76
*WT1* ^b^				
Negative	1.00 (reference)		1.00 (reference)	
Positive	1.48 (0.59–3.67)	0.40	1.22 (0.63–2.37)	0.55
*EVI1* ^b^				
Negative	1.00 (reference)		1.00 (reference)	
Positive	3.11 (0.70–13.76)	0.13*	1.27 (0.56–2.90)	0.57
*IgH* rearrangement				
No	1.00 (reference)		1.00 (reference)	
Yes	1.12 (0.13–9.45)	0.92	0.67 (0.15–2.88)	0.59
*TCR* rearrangement				
No	1.00 (reference)		1.00 (reference)	
Yes	0.63 (0.07–5.83)	0.68	1.65 (0.18–15.04)	0.66
Switched from CML lymphoblastic crisis				
No	1.00 (reference)		1.00 (reference)	
Yes	0.62 (0.12–3.08)	0.56	0.95 (0.24–3.79)	0.94
Disease status				
Relapsed	1.00 (reference)		1.00 (reference)	
Refractory	1.82 (0.60–5.56)	0.29	0.59 (0.29–1.19)	0.14*
Response after first chemotherapy		0.99		0.13*
CR	1.00 (reference)		1.00 (reference)	
PR	1.12 (0.24–5.29)	0.88	2.76 (0.60–12.65)	0.19*
NR	1.00 (0.32–3.16)	1.00	0.56 (0.25–1.26)	0.16*
Previous allo-HSCT				
No	1.00 (reference)		1.00 (reference)	
Yes	0.64 (0.26–1.58)	0.34	1.50 (0.69–3.28)	0.31
Number of relapses		0.04*		0.03*
0	1.00 (reference)		1.00 (reference)	
1	0.59 (0.16–2.19)	0.43	2.28 (1.06–4.92)	0.04*
>1	0.24 (0.06–0.91)	0.04*	0.97 (0.42–2.27)	0.95
Number of previous therapies		0.85		0.57
<3	1.00 (reference)		1.00 (reference)	
3–4	0.89 (0.32–2.51)	0.83	0.70 (0.33–1.49)	0.35
≥5	0.75 (0.27–2.07)	0.58	0.96 (0.44–2.10)	0.91

r/r B-ALL, refractory or relapsed B-cell acute lymphoblastic leukemia; CR, complete remission; MRD, minimal residual disease; OR, odds ratio; 95% CI, 95% confidence interval; WBC, white blood cells; CNS, central nervous system; Ph, Philadelphia chromosome; CML, chronic myeloid leukemia; PR, partial remission; NR, no response; allo-HSCT, allogeneic hematopoietic stem cell transplantation.

*Statistically significant (p < 0.2 for univariate analysis).

^#^EMD other than CNS involvement.

^a^The numbers of WBC in peripheral blood were detected when newly diagnosed.

^b^Positive: copy number>10/10,000abl copies, otherwise negative.

**Table 3:** Univariate logistic regression analyses of the baseline information of CAR-T therapy and CAR-T cells associated with CR and MRD-negative CR.

Variables	CR		MRD-negative CR	
	OR (95% CI)	*p* value	OR (95% CI)	*p* value
Bone marrow blasts (%) ^a^		0.01*		0.08*
<5	1.00 (reference)		1.00 (reference)	
5–25	0.48 (0.16–1.50)	0.21	0.73 (0.32–1.69)	0.46
25–50	0.82 (0.20–3.30)	0.78	0.60 (0.25–1.46)	0.26
≥50	0.19 (0.07–0.53)	<0.01*	0.34 (0.15–0.77)	0.01*
Lymphodepletion regimen				
Without FC	1.00 (reference)		1.00 (reference)	
FC	1.43 (0.29–6.98)	0.66	2.14 (0.63–7.32)	0.22
Decitabine				
No	1.00 (reference)		1.00 (reference)	
Yes	0.78 (0.16–3.78)	0.76	2.11 (0.45–9.94)	0.34
Rituximab				
No	1.00 (reference)		1.00 (reference)	
Yes	0.45 (0.11–1.77)	0.25	1.24 (0.32–4.74)	0.32
MVP				
No	1.00 (reference)		1.00 (reference)	
Yes	0.56 (0.17–1.82)	0.34	0.67 (0.25–1.80)	0.43
Infusion strategy		0.18*		0.18*
Single target	1.00 (reference)		1.00 (reference)	
Dual-target	3.94 (0.89–17.36)	0.07*	1.74 (0.78–3.93)	0.18*
Sequential infusion	1.77 (0.22–14.61)	0.60	4.28 (0.53–34.75)	0.17*
IL-6 knockdown				
No	1.00 (reference)		1.00 (reference)	
Yes	0.70 (0.14–3.42)	0.66	0.70 (0.20–2.48)	0.58
Generation				
2nd	1.00 (reference)		1.00 (reference)	
3rd or 4th	3.31 (0.96–11.44)	0.06*	2.01 (0.96–4.45)	0.06*
Glucocorticoid				
No	1.00 (reference)		1.00 (reference)	
Yes	0.66 (0.27–1.63)	0.37	0.59 (0.29–1.19)	0.14*
Tocilizumab				
No	1.00 (reference)		1.00 (reference)	
Yes	0.62 (0.21–1.82)	0.39	0.50 (0.22–1.17)	0.11*
CRS grade				
0–2	1.00 (reference)		1.00 (reference)	
>2	0.46 (0.20–1.05)	0.06*	0.42 (0.22–0.82)	0.01*
CRES				
0–2	1.00 (reference)		1.00 (reference)	
>2	0.34 (0.11–1.04)	0.06*	0.42 (0.15–21.15)	0.09*
Hemophagocytic histiocytosis				
No	1.00 (reference)		1.00 (reference)	
Yes	0.47 (0.05–4.67)	0.52	1.22 (0.12–12.02)	0.86
Tumor lysis syndrome				
No	1.00 (reference)		1.00 (reference)	
Yes	0.31 (0.03–3.54)	0.35	0.20 (0.02–2.23)	0.19*

CAR-T, chimeric antigen receptor T-cell immunotherapy; CR, complete remission; MRD, minimal residual disease; OR, odds ratio; 95% CI, 95% confidence interval; allo-HSCT, allogeneic hematopoietic stem cell transplantation; FC, fludarabine and cyclophosphamide; MVP, mitoxantrone, vincristine and prednisone; CRS, cytokine release syndrome; CRES, CAR-T cell-related encephalopathy syndrome.

*Statistically significant (p < 0.2 for univariate analysis).

^a^Bone marrow blasts detected before lymphodepletion or CAR-T cell infusion (for those without lymphodepletion).

### Identification of the Independent Factors Impacting CAR-T Therapeutic Response

In order to further identify the independent factors of the remission after CAR-T therapy, the possible influence factors (univariate logistic *p* < 0.2) from the above univariate analyses were incorporated into the following multivariate logistic regression analyses. The multivariate analytic results showed that the number of WBC in peripheral blood detected at diagnosis, CNS leukemia, *TP53* mutation, bone marrow blasts detected before lymphodepletion, or CAR-T cell infusion and CAR-T cell generation were significant independent factors of CR (*p* < 0.05, **Supplementary Table 1**), while for MRD-negative CR, the independent factors were as follows: disease status (refractory or relapsed disease), bone marrow blasts detected before lymphodepletion, or CAR-T cell infusion and infusion strategy which referred to the choice of the infusion of single- or dual-target CAR-T cells or sequential infusion of two single-specific CAR-T cells (*p* < 0.05, **Supplementary Table 2**).

### Construction of the Predictive Models for the Treatment Response of r/r B-ALL Patients Receiving CAR-T Cell Infusion

The models for predicting the probability of CR and MRD-negative CR of r/r B-ALL patients after CAR-T therapy were constructed based on the above stepwise multivariate logistic regression analysis. The predictive model for CR was as follows:


$${\text{P}}^{CR}=1/(1+e^{-\text{X}})$$

X=2.04−0.68×WBC−1.40×CNS−1.82×TP53−0.44×Blast+1.68×Generation

Definition and value:

P*^CR^* = the probability of CR;

X = the probability score of CR;

WBC: the number of WBC in peripheral blood detected at diagnosis; the value of WBC (×10^9^/L): <20:1; 20–100:2; ≥100:3;

CNS leukemia: no:0; yes = 1;

*TP53* mutation: no:0; yes = 1;

Blast: bone marrow blasts detected before lymphodepletion or CAR-T cell infusion (for those without lymphodepletion); the value of blast: <5:0; 5–25:1; 25–50:2; ≥50:3;

Generation: CAR-T cell generation used for this infusion; the value of generation: 2nd:1; 3rd or 4th:2.

The predictive model for MRD-negative CR was as follows:


$${\text{P}}^{MRD-CR}=1/(1+e^{-\text{Y}})$$

Y=0.56−0.77×Disease_status−0.47×Blast+0.83×Infusion_strategy

Definition and value:

P*^MRD-CR^* = the probability of MRD-negative CR;

Y = the probability score of MRD-negative CR;

Disease_status: relapsed:0; refractory:1;

Blast: bone marrow blasts detected before lymphodepletion or CAR-T cell infusion (for those without lymphodepletion); the value of Blast: <5:0; 5–25:1; 25–50:2; ≥50:3;

Infusion_strategy: infusion of single- or dual-target CAR-T cells or sequential infusion of two single-specific CAR-T cells; single target:1; dual-target:2; sequential infusion: 3.

**Figure 2:**
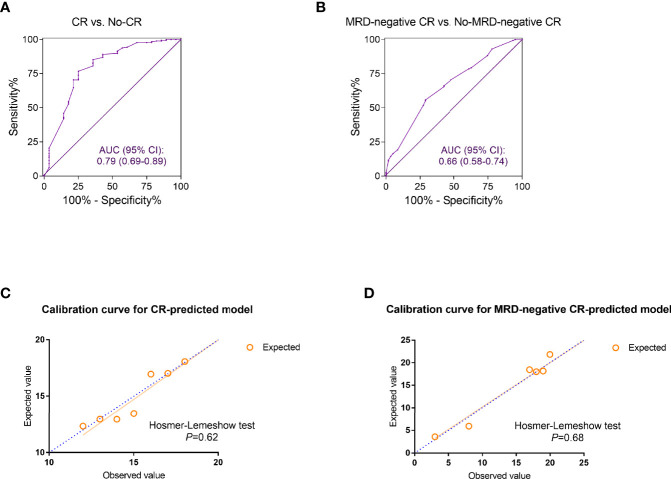
The discrimination and calibration evaluation of the complete remission (CR) and minimal residual disease (MRD)-negative CR predicted models. **(A, B)** The receiver operating characteristic (ROC) curves discriminating the refractory or relapsed (r/r) B-cell acute lymphoblastic leukemia (ALL) patients who achieved CR **(A)** or MRD-negative CR **(B)** from those who did not. The area under the curve (AUC) of ROC was calculated for evaluation. **(C, D)** Calibration curves for the estimation of CR **(C)** and MRD-negative CR **(D)**. The observed and the model-expected numbers of events (CR and MRD-negative CR) were plotted on the x- and y-axes, respectively. *p* values generated from Hosmer–Lemeshow tests were also calculated for assessing the goodness of fit of the constructed models.

The discrimination ability of the established models was assessed by ROC curves. The CR-predicting model exhibited great performance in distinguishing CR patients from those not achieving CR with the AUC reaching 0.79 (95% CI: 0.69–0.89) (**Figure 2A**). The AUC of the ROC curves plotted based on the predictive model for MRD-negative CR was 0.66 (95% CI: 0.58–0.74) (**Figure 2B**). The accuracy of the two models was evaluated by calibration curves. The calibration plots showed good agreement when comparing the expected values generated from the constructed models and the observed values. The Hosmer–Lemeshow tests of the two models showed great goodness of fit with the *p* values, 0.62 and 0.68, respectively (**Figures 2C, D**).

### Development and Preliminary Validation of the Constructed Predictive Models

**Figure 3:**
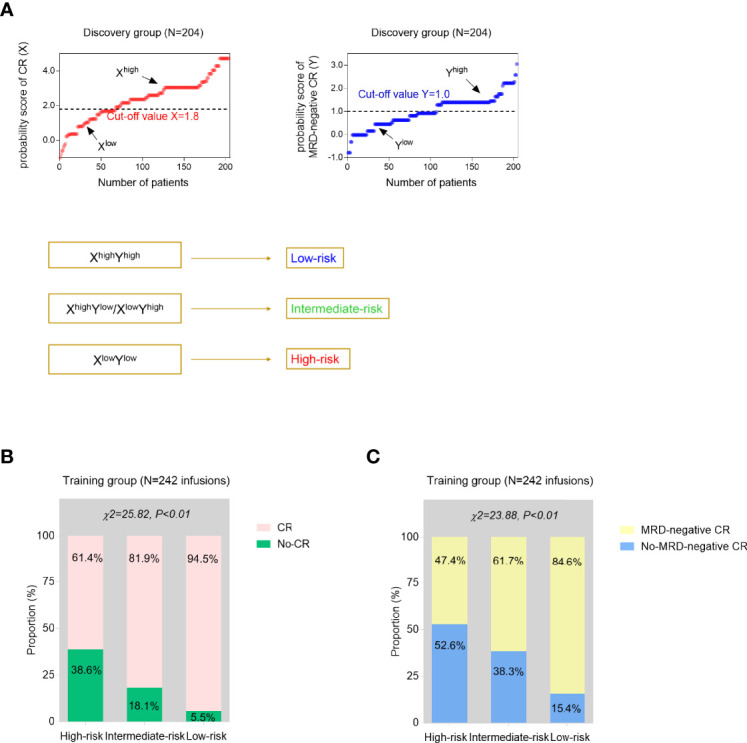
Development and validation analyses of the established models. **(A)** Using the CR-predicted and MRD-negative CR-predicted models, each time of CAR-T cell infusion could be evaluated as high probability of CR (X^high^) or low probability of CR (X^low^), high probability of MRD-negative CR (Y^high^), or low probability of MRD-negative CR (Y^low^) with the cutoff values generated from the discovery cohort. Then, two independent predictive models were further integrated as one risk stratification model based on the simultaneous estimation of the probability of CR and MRD-negative CR. **(B, C)** The proportions of CR **(B)** and MRD-negative CR **(C)** infusions were compared among three risk groups from the training group. *p* values were calculated *via* the chi-square (χ^2^) test.

To better predict the treatment response of r/r B-ALL patients after CAR-T therapy, the above two predictive models were further modified and integrated as one risk model. In the validation analyses, the training group was composed of the 242 infusions which 204 patients had altogether. A total of 242 infusions were divided into X^high^ and X^low^ groups, Y^high^ and Y^low^ groups, by the cutoff value, 1.8 and 1.0, respectively. The treatment outcome of an infusion evaluated as X^high^ and Y^high^ simultaneously was defined as the low-risk infusion while an infusion assessed as X^low^ and Y^low^ was defined as high-risk, otherwise intermediate-risk (**Figure 3A**). The CR and MRD-negative CR rates were compared among three subgroups (high-risk, intermediate-risk, and low-risk groups). The results showed that three risk groups had significantly different remission proportions (*p* < 0.01, **Figures 3B, C**).

**Figure 4:**
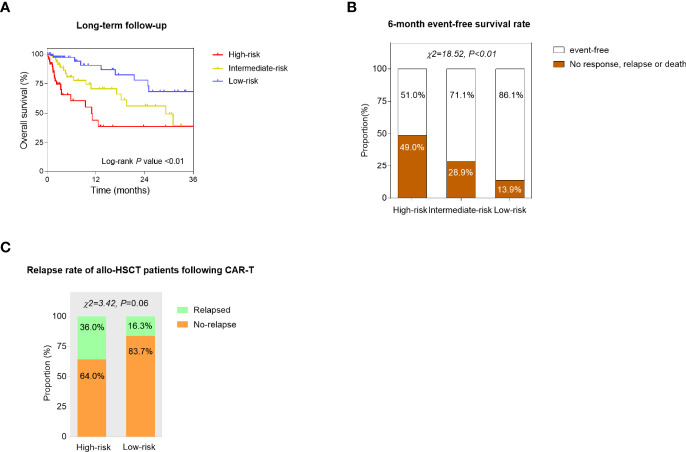
Confirmation of the prognostic value of the constructed models. **(A)** Kaplan–Meier curves of the high-risk, intermediate-risk, and low-risk r/r B-ALL patients. The overall survival (OS) was compared by the log-rank test. **(B)** The 6-month event-free survival (EFS) rate was compared among high-risk, intermediate-risk, and low-risk groups using the chi-square (χ^2^) test. **(C)** The relapse rate of the patients who received allogeneic hemopoietic stem cell transplantation (allo-HSCT) following CAR-T therapy was compared between high-risk and low-risk subgroups. *p* values were generated from the χ^2^ test.

To further explore the prognostic value of the predictive models, the long-term survival was compared among three risk groups. As seen from the Kaplan–Meier curves, low-risk patients had significantly better OS than high-risk and intermediate-risk patients (log-rank *p* < 0.01, **Figure 4A**). The 6-month EFS rate was also compared and significantly differed among three subgroups (*p* < 0.01, **Figure 4B**). In addition, the prognosis of the patients who received allo-HSCT after CAR-T cell infusion was assessed. 25 high-risk patients and 43 low-risk patients underwent allotransplant following CAR-T therapy altogether. The relapse rate in high-risk patients was higher than that in low-risk ones (*p* = 0.06, *p* value was insignificant probably because of the limited number of samples) (**Figure 4C**).

### Further Validation of the Risk Model Using the Independent Patient Group

**Figure 5:**
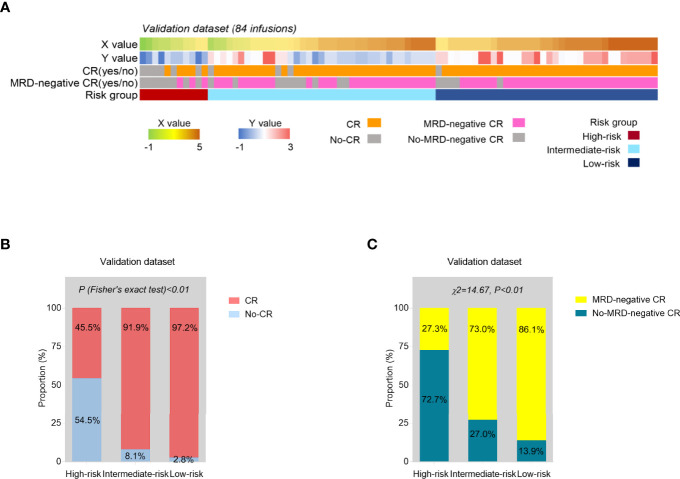
Internal validation of the risk model. **(A)** The predicted probability score of CR (X) and MRD-negative CR (Y) and risk group based on the constructed models, and the actual treatment response of each time of CAR-T cell infusion in the validation series (N = 84). **(B, C)** The proportions of CR **(B)** and MRD-negative CR **(C)** infusions were compared among three risk groups from the validation set. *p* values were calculated *via* Fisher’s exact test **(B)** and chi-square (χ^2^) test **(C)**.

The independent validation cohort consisted of 82 r/r B-ALL patients enrolled during a subsequent period of time. 82 patients had 84 infusions altogether (2 patients had twice). The baseline characteristics of the validation group are shown in **Supplementary Table 3**. According to the established predictive models for CR and MRD-negative CR, each time of CAR-T cell infusion could get an X and Y value. Then, based on the final risk model, all the 84 infusions (as the final validation dataset) could be stratified into high-risk, intermediate-risk, and low-risk groups (**Figure 5A**). The lower risk series showed higher CR and MRD-negative rates after CAR-T therapy, and the *p* value was statistically significant (*p* < 0.01, **Figures 5B, C**).

## Discussion

In this study, we constructed the risk model of both predictive and prognostic values in r/r B-ALL patients receiving CAR-T therapy. Our final model integrated two independent predicting models for CR and MRD-negative CR as a second modification which can be expected of better predictive efficiency. Whether the integration model or the independent models were easy to use because all used variables could be conveniently obtained before the treatment, as they were all routine tests and baseline clinical data. Moreover, the contribution of each variable to the whole model could be quickly calculated according to their coefficients in the formulas which greatly increased its clinical applicability as well. Using the models, r/r B-ALL patients could be stratified into different risk groups, predicted of the treatment response in advance of CAR-T cell infusion, and provided with personalized therapeutic advice.

Consistent with the previous studies, our results also showed that high tumor burden especially marrow disease burden was related to poor treatment response of CAR-T therapy in r/r B-ALL patients (17, 19, 29). Multivariate analytic results demonstrated that the number of bone marrow blasts before lymphodepletion or CAR-T cell infusion was the independent factor of both CR and MRD-negative CR. The other direct marker of tumor burden, the number of WBC in peripheral blood when newly diagnosed, was an independent factor of CR. From univariate analysis, WBC ≥ 100 × 10^9^/l was a significant risk factor (*p* = 0.04) of MRD-negative CR although it was not independently associated with MRD-negative CR. Therefore, pretreatment to lower leukemia burden particularly for those high-risk patients before infusion with CAR-T cells might be a cardinal approach to improve CAR-T therapeutic effects.

In terms of the other baseline characteristics of r/r B-ALL patients, our findings showed that *TP53* mutation and CNS leukemia were the independent risk factors of CR. It has been demonstrated by numerous studies that *TP53* mutation was associated with unfavorable outcome of patients with various cancers including hematological malignancies (30, 31). A recent large-cohort retrospective study by Zhang et al. also reported that *TP53* mutation was independently correlated with the CR rate in B-ALL patients receiving CD19 CAR-T therapy (17). A non-randomized clinical trial involving 51 r/r B-ALL participants receiving CD19 CAR-T cells showed that extramedullary disease (EMD) other than CNS leukemia was independently associated with the poor survival of B-ALL patients after CAR-T therapy (18). However, in our study, we did not find significant relations between EMD and CR or MRD-negative CR, which was in accord with the results of Professor Zhang and her colleagues’ study (17). Anyway, these risk factors might greatly influence CAR-T treatment response and r/r B-ALL patients harboring these factors were probably not the appropriate candidates for the therapy.

Interestingly, we found that disease status (relapsed or refractory) right before infusion was the independent factor of MRD-negative CR in r/r B-ALL patients. Relapsed patients had a higher probability of MRD-CR remission after CAR-T therapy than refractory ones. To the best of our knowledge, clinical and experimental evidence is lacking in comparing the treatment response of these two subgroups at present. Our findings in this study led to speculation that relapsed patients might be more sensitive to antitumor therapy while refractory patients were more easily resistant to the treatment. However, more studies are needed to validate our results and reveal molecular biological mechanisms.

With respect to the characteristics of CAR-T cells used for each infusion, our study revealed that the third and fourth generations of CAR-T cells were associated with higher probability of remission compared to the second generation. Besides, for target recognition, either dual-target infusions or sequential infusions of two single-specific CAR-T cells demonstrated better clinical response than single-target infusions. At present, dual-target infusion is considered as a promising and effective strategy for the antigen-loss relapse after single-target CAR-T cells (32). A previous meeting abstract and a case report showed that sequential infusions could significantly improve the clinical outcome of r/r B-ALL patients as well (33, 34). Recently, sequential infusion of two single-specific CAR-T cells was proposed as a cocktail therapy in r/r B-ALL patients for its proved superior efficacy and safety in the clinical trial (35), while thus far, few clinical trials comparing the efficacy of dual-target and sequential infusions have been reported. In our study, we found that patients who received sequential infusions were more likely to achieve MRD-negative CR compared to those after dual-target infusions. However, more targets or latest generation indicated higher cost of this therapy. Accordingly, in order to reduce treatment expenses of patients, prioritize medical resources, and meanwhile, improve individual prognosis, based on our models, low-risk patients are highly recommended for latest and dual-targeting or sequential infusion of CAR-T cells.

Our study has several limitations. Firstly, selection bias of the retrospective study was inevitable. Secondly, although the patients were from three clinical trials, they were treated in one similar medical center and were all Chinese. Therefore, larger patient groups, especially non-Chinese populations, from different medical centers are needed to further validate our models. Moreover, the application of our risk model was finally extended to each time of CAR-T cell infusion. However, the sample size of other infusions (not first-time infusion) was limited in this study. Thus, more real-world data of multiple CAR-T cell infusions of one single patient are need for validation as well. Besides, our models were constructed based on several common types of CAR-T cells. To further broaden the application of these models, more data of patients receiving the CAR-T cells of different targets, structures, and origins are needed to test and validate the models. Lastly, before putting into analyses, all the continuous variables were transformed into categorical ones to facilitate the application, which might simultaneously decrease the robustness of the constructed models. In spite of all the limitations, the predictive models for r/r B-ALL patients after CAR-T therapy, hereon we attempted to establish for the first time, displayed excellent clinical efficacy even in prognosis estimation.

In summary, we here developed and validated the predictive models for CAR-T therapeutic responses in r/r B-ALL patients and further confirmed the prognostic value of the risk model. The combined application of the two independent models for CR and MRD-negative CR estimation and the modified risk model can provide credible advice on the selection of the most benefited r/r B-ALL patients from this novel therapy and on the prevention treatment of these patients for clinicians and hematologists. What is more, as commercial products of CAR-T cells are increasingly reported these years, our models need to be tested and improved based on these multicenter real-world data. Nevertheless, our findings may also give clinical implications in improving CAR-T therapeutic effectiveness.

## Data Availability Statement

The raw data supporting the conclusions of this article will be made available by the authors, without undue reservation.

## Ethics Statement

The studies involving human participants were reviewed and approved by the Institutional Ethics Committees of the First Affiliated Hospital of Soochow University. Written informed consent to participate in this study was provided by the participants’ legal guardian/next of kin.

## Author Contributions

XT, DW, and JG designed the research. JG, SL, HD, QC, JY, ZL, LK, HQ, YH, MM, SC, SX, YW, ZJ, XZ, and LY cared for the enrolled patients and conducted the medical visits. LK and LY were also responsible for the production of CAR-T cells. JG, WC, and SL collected and analyzed the patients’ data. HD, QC, JY, and ZL constructed the figures. JG and WC drafted the manuscript. XT and DW revised the manuscript. All authors contributed to the article and approved the submitted version.

## Funding

This work was supported by research grants from the National Natural Science Foundation of China (81873443, 82070162, 81900175, 81400155, 81700139), Major Natural Science Research Projects in institutions of higher education of Jiangsu Province (19KJA210002), The Key Science Research Project of Jiangsu Commission of Health (K2019022), Translational Research Grant of NCRCH (2020ZKZC04), Natural Science Foundation of Jiangsu Province (BK20190181, BK20201169, BK20170360), the Frontier Clinical Technical Project of the Science and Technology Department of Jiangsu Province (BE2018652), and the Priority Academic Program Development of Jiangsu Higher Education Institutions (PAPD).

## Conflict of Interest

Authors LK and LY are employed by Shanghai Unicar-Therapy Bio-Medicine Technology Co., Ltd.

The remaining authors declare that the research was conducted in the absence of any commercial or financial relationships that could be construed as a potential conflict of interest.

## Publisher’s Note

All claims expressed in this article are solely those of the authors and do not necessarily represent those of their affiliated organizations, or those of the publisher, the editors and the reviewers. Any product that may be evaluated in this article, or claim that may be made by its manufacturer, is not guaranteed or endorsed by the publisher.
