# Supplementary material for: Identification of the Predictive Models for the Treatment Response of Refractory/Relapsed B-Cell ALL Patients Receiving CAR-T Therapy
Source: Front Immunol. 2022 Mar 17;13:858590. doi: 10.3389/fimmu.2022.858590 (PMC8970344; doi:10.3389/fimmu.2022.858590)
Supplement: Supplementary file 1 [file Table_1.docx]

**Supplementary table 1. The identified independent factors of CR from multivariate Logistic regression analysis.**

| **Variables** | **B*** | **Standard error** | **Wald** | **Degree of freedom** | ***P* value** | **OR (95% CI)** |
| --- | --- | --- | --- | --- | --- | --- |
| WBC | -0.68 | 0.27 | 6.20 | 1 | 0.01 | 0.50(0.30-0.86) |
| CNS leukemia | -1.40 | 0.67 | 4.37 | 1 | 0.04 | 0.25(0.07-0.92) |
| *TP53* mutation | -1.82 | 0.65 | 7.89 | 1 | <0.01 | 0.16(0.04-0.58) |
| Blast | -0.44 | 0.20 | 5.19 | 1 | 0.02 | 0.64(0.44-0.94) |
| Generation | 1.68 | 0.73 | 5.27 | 1 | 0.02 | 5.36(1.28-22.49) |
| Constant | 2.04 | 0.97 | 4.48 | 1 | 0.03 | 7.72 |

Abbreviations: CR, complete remission; OR, odds ratio; 95% CI, 95% confidence interval; WBC: white blood cells (the number of WBC in peripheral blood detected when newly diagnosed); CNS, central nervous system; Blast: bone marrow blasts detected before lymphodepletion or CAR-T cell infusion (for those without lymphodepletion); Generation: CAR-T cell generation used for this infusion.

*: coefficients for variables.
