# Supplementary material for: Identification of the Predictive Models for the Treatment Response of Refractory/Relapsed B-Cell ALL Patients Receiving CAR-T Therapy
Source: Front Immunol. 2022 Mar 17;13:858590. doi: 10.3389/fimmu.2022.858590 (PMC8970344; doi:10.3389/fimmu.2022.858590)
Supplement: Supplementary file 2 [file Table_2.docx]

**Supplementary table 2. The identified independent factors of MRD-negative CR from multivariate Logistic regression analysis.**

| **Variables** | **B*** | **Standard error** | **Wald** | **Degree of freedom** | ***P* value** | **OR (95% CI)** |
| --- | --- | --- | --- | --- | --- | --- |
| Disease status | -0.77 | 0.38 | 4.09 | 1 | 0.04 | 0.46(0.22-0.98) |
| Blast | -0.47 | 0.14 | 10.71 | 1 | <0.01 | 0.62(0.47-0.83) |
| Infusion strategy | 0.83 | 0.34 | 5.82 | 1 | 0.02 | 2.30(1.17-4.51) |
| Constant | 0.56 | 0.46 | 1.45 | 1 | 0.23 | 1.74 |

Abbreviations: MRD: minimal residual disease; CR, complete remission; OR, odds ratio; 95% CI, 95% confidence interval; Blast: bone marrow blasts detected before lymphodepletion or CAR-T cell infusion (for those without lymphodepletion).

*: coefficients for variables.
