# Supplementary material for: Identification of the Predictive Models for the Treatment Response of Refractory/Relapsed B-Cell ALL Patients Receiving CAR-T Therapy
Source: Front Immunol. 2022 Mar 17;13:858590. doi: 10.3389/fimmu.2022.858590 (PMC8970344; doi:10.3389/fimmu.2022.858590)
Supplement: Supplementary file 3 [file Table_3.docx]

**Supplementary table 3. Baseline characteristics of the validation cohort (N=82).**

| **Characteristics^#^** | **n*** |
| --- | --- |
| Gender (female/male) | 44/38 |
| Age (years) | 36 (12-65) |
| WBC^a^ (×10^9/L) (<20/20-100/≥100) | 30/29/23 |
| CNS leukemia (yes/no) | 6/76 |
| Ph+ (yes/no) | 36/46 |
| *TP53* mutation (yes/no) | 6/76 |
| Disease status (relapsed/refractory) | 47/35 |
| Previous allo-HSCT (yes/no) | 14/68 |
| Bone marrow blasts (%) ^b^ (<5/5-25/25-50/>50) | 40/24/9/9 |
| Infusion strategy (single target/dual-target/sequential infusion) | 44/13/25 |
| Generation (2^nd^/3^rd^ or 4^th^) | 42/40 |

Abbreviations: WBC: white blood cells; CNS, central nervous system; Ph: Philadelphia chromosome; allo-HSCT, allogeneic hematopoietic stem cell transplantation;

^#^: For two patients who received more than one time of CAR-T cell infusion, only the baseline information of the first infusion was presented.

*: Median and range for age, absolute patient numbers for other covariates.

^a^: The numbers of WBC in peripheral blood were detected when newly diagnosed;

^b^: Bone marrow blasts detected before lymphodepletion or CAR-T cell infusion (for those without lymphodepletion).
